# Comparison of human B cell activation by TLR7 and TLR9 agonists

**DOI:** 10.1186/1471-2172-9-39

**Published:** 2008-07-24

**Authors:** John A Hanten, John P Vasilakos, Christie L Riter, Lori Neys, Kenneth E Lipson, Sefik S Alkan, Woubalem Birmachu

**Affiliations:** 1 Department of Pharmacology, 3M Pharmaceuticals, St. Paul, MN 55144, USA; 2 3M Drug Delivery Systems, St. Paul, MN 55144, USA; 3 Biothera, 3388 Mike Collins Dr, Eagan, MN 55121, USA; 4 DiaSorin, 1951 Northwestern Ave, P.O. Box 285, Stillwater MN 55082, USA; 5 FibroGen, Inc., 225 Gateway Blvd., South San Francisco, CA 94080, USA; 6 Alba Therapeutics Corp, 800 W. Baltimore St., Suite 400, Baltimore, MD 21201, USA; 7 3M Medical, St. Paul, MN 55144, USA

## Abstract

**Background:**

Human B cells and plasmacytoid dendritic cells (pDC) are the only cells known to express both TLR7 and TLR9. Plasmacytoid dendritic cells are the primary IFN-α producing cells in response to TLR7 and TLR9 agonists. The direct effects of TLR7 stimulation on human B cells is less understood. The objective of this study was to compare the effects of TLR7 and TLR9 stimulation on human B cell function.

**Results:**

Gene expression and protein production of cytokines, chemokines, various B cell activation markers, and immunoglobulins were evaluated. Purified human CD19^+ ^B cells (99.9%, containing both naïve and memory populations) from peripheral blood were stimulated with a TLR7-selective agonist (852A), TLR7/8 agonist (3M-003), or TLR9 selective agonist CpG ODN (CpG2006). TLR7 and TLR9 agonists similarly modulated the expression of cytokine and chemokine genes (IL-6, MIP1 alpha, MIP1 beta, TNF alpha and LTA), co-stimulatory molecules (CD80, CD40 and CD58), Fc receptors (CD23, CD32), anti-apoptotic genes (BCL2L1), certain transcription factors (MYC, TCFL5), and genes critical for B cell proliferation and differentiation (CD72, IL21R). Both agonists also induced protein expression of the above cytokines and chemokines. Additionally, TLR7 and TLR9 agonists induced the production of IgM and IgG. A TLR8-selective agonist was comparatively ineffective at stimulating purified human B cells.

**Conclusion:**

These results demonstrate that despite their molecular differences, the TLR7 and TLR9 agonists induce similar genes and proteins in purified human B cells.

## Background

B lymphocytes play an essential role in bridging innate and adaptive immunity. Through ligand receptor signaling they differentiate into specialized cells capable of communicating with helper T cells in order to undergo antibody diversification, clonal expansion and immunoglobulin secretion. Various ligands and their corresponding receptors are responsible for these signaling events leading towards B cell activation and maturation. Among recently discovered B cell activators, of particular interest are the Toll-like receptors (TLRs) and their natural agonists responsible for eliciting direct effects on human B cells. Natural TLR agonists have been shown to elicit an innate immune response in human blood leukocytes including peptidoglycan and lipoproteins (TLR2), dsRNA, polyI:C (TLR3), LPS (TLR4), flagellin (TLR5), guanosine and uridine rich ssRNA (TLR7), and oligodeoxynucleotides (ODNs) with CpG motifs (TLR9) [1-5]. The Immune Response Modifier (IRM) Imiquimod (R-837) has been shown to activate NF-κB through TLR7 while Resiquimod (R-848) has been shown to activate NF-κB through TLR7 and TLR8 [6,7]. Plasmacytoid dendritic cells express TLR7 and TLR9, and are the main type 1 interferon producing cells in response to IRMs and CpGs, respectively [8-10]. B cells are the only other human leukocyte subset to express both TLR7 and TLR9, and have also been shown to be directly activated by IRMs and CpGs [11-14]. It has been reported that memory and naïve human B cells differentially respond to TLR7 and TLR9 stimulation, with type I IFN being required for TLR7-mediated polyclonal B cell expansion, TLR7 up-regulation, and B cell differentiation towards immunoglobulin-producing plasma cells, but not for TLR9-mediated B cell activation [15].

The objective of this study was to compare and contrast the effects of TLR7- and TLR9-mediated B cell activation by examining changes in gene and protein expression in purified human B cells. The B cell population used in these studies contained both naïve and memory populations of cells but was devoid of pDC. The results demonstrate that CD19^+ ^B cells isolated from peripheral blood similarly respond to TLR7 and TLR9 stimulation in regard to cytokine and chemokine expression as well as expression of selected co-stimulatory markers, Fc receptors, anti-apoptotic genes, transcription factors, and differentiation and proliferation genes.

## Results

### B cell purity and TLR basal gene expression

**Figure 1:**
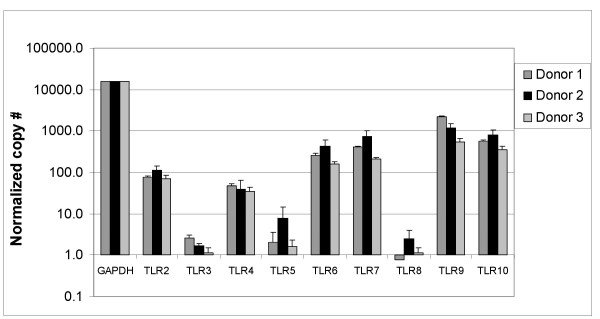
**Relative levels of TLR2 to TLR10 mRNA expression in human B cells from 3 different donors.** Highly purified B cells from 3 different donors were examined for expression of the TLRs by RT-PCR. The copy number for TLR2 to TLR10 mRNA was normalized to that for GAPDH to compare expression between donors.

B cells were enriched from human PBMC by negative selection and then purified by cell sorting. Prior to sorting, the enriched B cell population was about 80% pure, and the final purity after sorting was ≥99% (see Additional file 1). The expression of Toll-like receptors (TLR) in purified B cells from 3 donors was determined by RT-PCR (Figure 1) and quantitated using the ΔΔCt method [16]. The B cells expressed intermediate to high levels of TLR6, TLR7, TLR9, and TLR10, and about 10-fold lower levels of TLR2 and TLR4. The expression levels of TLR3, TLR5, and TLR8 were at the lower limit of detection for the assay. The TLR expression profiles from the 3 different donors were similar, and are consistent with previously published studies [17,18]. The levels of TLR1 mRNA were not measured in this study.

### Characterization of small molecule TLR7, TLR7/8, and TLR8 agonists

The potency and TLR7 *vs. *TLR8 selectivity profiles of the IRMs used in this study were previously demonstrated [6,19]. At the concentrations used, 852A preferentially activates NF-κB through TLR7, 3M-002 preferentially activates NF-κB through TLR8, and 3M-003 activates NF-κB through both TLR7 and TLR8. For ease of discussion, throughout this paper, 852A will be referred to as a TLR7 agonist, 3M-002 will be referred to as a TLR8 agonist, 3M-003 will be referred to as a TLR7/8 agonist, and CpG2009 will be referred to as a TLR9 agonist. However, these names should not be construed as indicating absolute selectivity, since at higher concentrations, the preferential selectivity breaks down, and 852A can activate through TLR8 while 3M-002 can activate through TLR7. Therefore, as was reported in other systems [20], the concentration of test compound used must be carefully selected to observe and correctly interpret the desired effects. The minimum effective concentration required to activate NF-κB in TLR-transfected HEK293 cells is shown in Table 1, illustrating the TLR selectivity of the various small molecule TLR agonists at the concentrations used in this study.

**Table 1:** Potency of small molecule TLR agonists in activating TLR7- and TLR8-mediated NF-κB activity in transfected human HEK293 cells.

**Compound**	**Minimum effective concentration (MEC, μM) for NFκB activation (a)**		**TLR selectivity (b)**
		
	**TLR7**	**TLR8**	
**3M-006**	Inactive (100)	Inactive (100)	None
**3M-003**	0.1	1	TLR7/8 @ 1 μM
**852A**	3	100	TLR7 @ 3 μM
**3M-002**	10	1	TLR8 @ 5 μM

(a) Transfection of HEK293 cells with TLR7 or TLR8, and assessment of IRM-induced NFκB activation was performed as previously described [6]. Potency is defined as the concentration required to induce an increase in NFκB activation. MEC, minimal effective concentration, is the lowest concentration required to observe a 2-fold increase above the vehicle control.

(b) Selectivity is defined as preferential signaling through TLR7 or TLR8 at a specified concentration of the TLR agonists.

### TLR agonist induced gene expression

**Figure 2:**
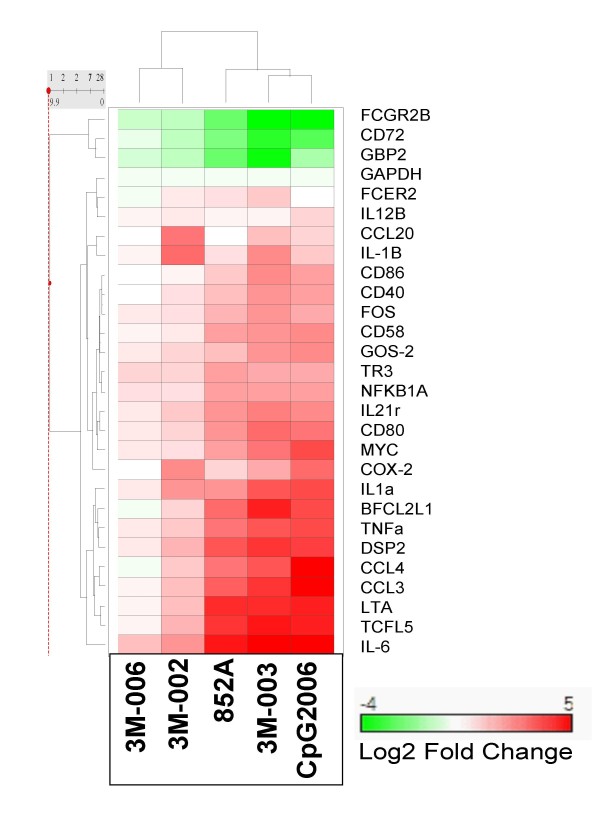
**Profile of gene expression changes in human B cells stimulated with agonists of TLR7, 8 or 9.** Purified B cells were stimulated with the indicated IRM or with CpG2006. Gene expression changes were assessed by quantitative real time RT-PCR at 2, 8 or 24 hours after stimulation. The log2 of the maximum fold change over the time course from 2 to 24 hours for 1 representative donor is shown. Hierarchical clustering was performed as described in methods.

The expression profiles of cytokine, chemokine, proliferation, differentiation, co-stimulatory, Fc receptor, transcription factor, and anti-apoptotic genes were evaluated in purified B cells at 2, 8, and 24 hr following stimulation with TLR agonists. The time course of gene expression for one representative donor is illustrated (see Additional file 2 and Additional file 3). Gene expression was predominantly maximal at either 2 or 8 hours post treatment. The maximum gene expression for one representative donor is illustrated in Figure 2 as a heat map. The maximum gene expression data for all 3 donors are summarized in Table 2.

**Table 2:** Gene expression profile of human B cells modulated by TLR7, 8, or 9 agonists from 3 different donors (maximum fold-change through a 2, 8, 24 hour time course).

**Gene**	**Alias**	**3M-006**			**3M-002**			**852A**			**3M-003**			**CpG 2006**		
		**D1**	**D2**	**D3**	**D1**	**D2**	**D3**	**D1**	**D2**	**D3**	**D1**	**D2**	**D3**	**D1**	**D2**	**D3**
**FCGR2B**	**CD32**	-1.2	-1.7	-1.2	-1.3	-1.9	-1.3	-2.6	**-4.7**	-2.1	**-7.4**	**-19.1**	**-8.4**	**-6.7**	**-22.8**	**-8.6**
**GBP2**	**GBP2**	-1.1	-1.5	1.0	-1.1	-1.9	1.0	-2.8	**-5.0**	-2.3	**-3.5**	**-13.4**	**-7.1**	-2.6	-1.7	-1.8
**CD72**	**Ly-19**	-1.5	1.2	1.4	-1.1	-1.2	1.4	-3.2	**-3.9**	-1.8	**-5.1**	**-9.1**	**-4.4**	**-3.5**	**-5.8**	-2.9
**GAPDH**	**GAPDH**	1.0*	1.0*	1.0*	1.0*	1.0*	1.0*	1.0*	1.0*	1.0*	1.0*	1.0*	1.0*	1.0*	1.0*	1.0*
**IL12B**	**IL12p40**	-1.1	1.3	1.2	**8.5**	1.5	1.1	**9.1**	-1.1	2.5	**16.1**	1.3	**3.7**	**11.4**	-1.2	1.6
**FCER2**	**CD23**	-1.1	-1.4	-1.3	1.6	1.4	1.5	3.1	1.7	2.9	**7.0**	2.3	**5.4**	**3.5**	1.1	**3.7**
**CCL20**	**MIP3a**	-1.2	-1.2	1.2	**52.3**	**6.7**	1.3	2.9	-1.6	1.0	**28.6**	2.6	1.4	-2.9	-2.1	1.0
**IL-1B**	**IL1F2**	-1.7	-1.6	-1.8	**31.1**	**8.2**	**29.1**	2.6	1.7	2.8	**28.8**	**5.4**	**9.8**	**3.9**	-1.7	3.2
**COX-2**	**PTGS2**	-1.2	-2.8	1.3	**21.7**	**5.0**	2.0	-2.7	2.0	2.7	**16.8**	**3.6**	**5.6**	**4.9**	**8.3**	2.2
**CD86**	**B7-2**	-1.2	-1.2	1.2	1.2	1.3	1.3	2.1	2.3	2.3	2.8	**5.0**	**4.1**	1.6	**3.8**	1.9
**CD40**	**TNFRSF5**	1.1	1.1	1.1	1.6	1.6	1.6	**3.7**	2.6	3.1	**6.1**	**4.7**	**6.0**	**4.7**	**4.0**	3.0
**FOS**	**c-fos**	1.1	1.4	1.6	-1.3	1.6	1.8	-2.0	2.8	2.7	**-3.7**	**4.7**	**6.1**	-2.7	-2.0	-1.6
**GOS-2**	**RP1**	-1.4	-1.4	2.0	2.3	2.0	2.6	3.1	2.7	**4.4**	**6.3**	**4.5**	**10.0**	**5.3**	**5.1**	**7.5**
**NFKB1A**	**IKBA**	1.1	1.5	1.4	1.9	1.7	2.2	**3.8**	**3.8**	**4.2**	**4.8**	**4.2**	**6.6**	**4.0**	**4.2**	**5.4**
**TR3**	**TNFRSF25**	1.3	1.8	1.6	1.2	1.9	2.1	1.5	**4.0**	**3.5**	-2.7	3.2	**5.6**	2.0	**3.6**	-1.5
**CD58**	**LFA3**	1.1	1.2	1.2	1.3	1.5	1.6	2.0	**3.8**	3.2	3.3	**4.8**	**5.0**	3.2	**5.2**	**5.5**
**IL21r**	**NILR**	1.3	1.4	1.3	1.7	2.2	2.2	**3.5**	**4.7**	**5.4**	**4.9**	**5.7**	**6.7**	**4.0**	**5.1**	**6.3**
**MYC**	**c-myc**	-1.2	1.4	1.4	1.6	1.7	1.9	2.1	**3.7**	3.2	**3.5**	**7.0**	**10.7**	**3.6**	**12.3**	**8.1**
**CD80**	**B7-1**	1.2	1.4	1.2	2.0	1.9	1.8	**3.9**	**4.8**	**3.8**	**7.3**	**8.6**	**8.1**	**6.1**	**6.9**	**6.4**
**TNFa**	**TNFSF2**	-1.2	1.4	1.2	3.1	2.2	1.7	**9.5**	**6.9**	**7.7**	**14.9**	**10.3**	**16.2**	**13.8**	**11.8**	**7.0**
**IL1a**	**IL1F1**	-1.7	1.5	1.2	2.5	**4.5**	1.5	**4.0**	**4.3**	2.8	**5.7**	**11.4**	**6.8**	**4.6**	**11.7**	2.9
**CCL4**	**MIP1b**	1.4	1.0	1.2	3.3	2.3	3.0	**6.1**	**7.1**	**5.7**	**10.8**	**10.9**	**32.6**	**22.3**	**38.6**	**37.4**
**CCL3**	**MIP1a**	1.2	1.2	1.5	**4.5**	2.4	2.9	**11.4**	**10.0**	**10.1**	**18.9**	**15.6**	**45.6**	**39.3**	**45.6**	**62.3**
**BFCL2L1**	**Bcl-xl**	-1.3	-1.1	1.3	1.9	1.8	2.1	**4.5**	**8.3**	**6.4**	**6.1**	**21.7**	**15.1**	**5.1**	**12.8**	**9.2**
**DSP2**	**PAC1**	1.3	1.5	1.2	1.2	2.8	2.0	2.3	**10.8**	**4.6**	**4.0**	**16.1**	**18.3**	**3.7**	**13.5**	**5.0**
**LTA**	**TNFSF1**	1.0	1.3	1.3	**6.1**	2.5	2.5	**63.0**	**18.8**	**24.3**	**85.8**	**18.5**	**32.0**	**55.5**	**22.1**	**24.9**
**TCFL5**	**E2BP1**	-1.2	1.3	1.2	1.6	3.1	2.0	**9.7**	**17.7**	**9.4**	**22.6**	**25.2**	**24.9**	**16.3**	**23.5**	**18.7**
**IL-6**	**IFNB2**	1.9	2.4	1.0	**7.0**	**4.7**	**6.1**	**24.9**	**25.6**	**40.4**	**70.8**	**51.3**	**71.9**	**58.9**	**59.4**	**53.2**

(a) normal text = subjectively assigned nominal to low fold change (-3.4 to 3.4).

(b) bold text, negative values = subjectively assigned moderate fold suppression (-23.0 to -3.5).

(c) bold text, positive values = subjectively assigned moderate to high fold increase (3.5 to 86.0).

(d) text with * = GAPDH, house keeping gene as a reference.

#### Cytokine, chemokine and pro-inflammatory mediator genes

In general, the gene expression profiles of B cells treated with 3 μM 852A, 1 μM 3M-003, or 3 μM CpG2006 were similar in regard to the specific genes that were modulated. Figure 2 shows a 2 way hierarchical clustering of the log 2 transformed fold change of genes altered in expression. The figure shows that 852A, 3M-003 and CpG2006 cluster together, indicating a similar gene expression profile. However, the magnitude of modulation for most genes was greater in B cells treated with the TLR7/8 agonist 3M-003 and the TLR9 agonist CpG2006, compared to those treated with 852A, at the tested concentrations. For example, CCL3 (MIP1α), CCL4 (MIP1β), and IL6 genes were 2 to 5 times higher in expression in 3M-003- or CpG2006-treated B cells as compared to those treated with 852A. In other instances, however, the levels of gene modulation by 852A, 3M-003, and CpG2006 were similar. For example, TNFα and TNFβ (Lymphotoxin alpha, LTA) genes were equally modulated by 852A, 3M-003 or CpG 2006 in treated B cells, where all three TLR agonists resulted in 3- to 5-fold more LTA than TNFα gene expression. Note that the inactive analog, 3M-006, which was denoted as inactive because it did not induce cytokine production from human PBMC in contrast to 3M-001, 3M-002, and 3M-003 [6], was comparatively inactive for all genes evaluated in this study.

Interestingly, some genes were modulated by the TLR8 agonist 3M-002, which is likely due to residual NF-κB activation through TLR7. In comparison to the magnitude of gene induction by TLR7, TLR7/8, or TLR9 agonists, the TLR8 agonist generally induced lower levels of the same genes. However, the exceptions were MIP3α, IL1β, and COX2, which were induced in at least 2/3 of the donors by the agonists that can activate NF-κB through TLR8 (3M-002 and 3M-003) but not by the TLR7-selective agonist 852A or the TLR9 agonist CpG2006. Based on the purity of the CD19^+ ^population, it is unlikely that cells known to respond to TLR8 agonists, such as monocytes or conventional DC, are responsible for the induction of these proinflammatory genes. However, we can not rule out the possibility that low numbers of contaminating monocytes or conventional (myeloid) DC were responsible for the induction of some genes, such as IL-1β, since 3M-002 strongly induces IL-1β production from these cells (6). Another possibility is that 3M-002 may drive production of cytokines like IL-1β through interaction with other systems such as the cryopyrin pathway.

Because TLR7 and TLR9 agonists are known to robustly stimulate expression of type I IFNs and IFN-inducible genes in pDC [8,9,21-24], it was essential to eliminate pDC from the cell preparation in order to characterize the effects of these TLR agonists on human B cells. To determine if functional pDC were contaminating the B cell population, the expression of type I IFN and IFN-inducible genes that are indicative of pDC activation (IFNα 2, MX1, ISG15, TLR7 and TLR9) were evaluated following stimulation with the TLR agonists (Table 3). None of these genes were consistently induced by the TLR agonists, indicating that functional pDC were not present in the B cell population. However, some low-level of inconsistent gene activation was observed between donors, which is probably due to variations in assay conditions. B cell secreted protein values, assayed by Luminex, also confirmed that there was no protein production of IFN alpha or the type I IFN-inducible IP10 (data not shown).

**Table 3:** Interferon and interferon-inducible genes in human B cells were minimally and inconsistently modulated by TLR7, 8, or 9 agonists from 3 different donors (maximum fold-change through a 2, 8, 24 hour time course).

**Gene**	**Alias**	**3M-006**			**3M-002**			**852A**			**3M-003**			**CpG 2006**		
		**D1**	**D2**	**D3**	**D1**	**D2**	**D3**	**D1**	**D2**	**D3**	**D1**	**D2**	**D3**	**D1**	**D2**	**D3**
**GAPDH**	**GAPDH**	1.0*	1.0*	1.0*	1.0*	1.0*	1.0*	1.0*	1.0*	1.0*	1.0*	1.0*	1.0*	1.0*	1.0*	1.0*
**IFNa-2**	**IFNa-2**	2.6	1.7	1.5	**3.5**	1.8	1.4	3.0	-1.3	1.2	-2.2	-1.5	-1.2	4.0	-1.7	-1.2
**ISG15**	**ISG15**	1.6	1.3	1.2	1.9	1.1	1.5	1.2	1.5	1.8	3.0	-2.7	2.3	-1.5	-2.2	-1.5
**MX1**	**MX1**	1.3	-1.2	1.3	1.7	-1.5	1.1	1.5	-2.3	-1.6	**6.8**	-3.3	**-4.2**	**-3.9**	-3.4	**-4.3**
**TLR7**	**TLR7**	1.5	-1.2	1.6	1.2	-1.3	1.3	-2.8	-2.7	-1.6	-2.9	-2.1	**-4.3**	**-3.5**	**-6.7**	**-4.5**
**TLR9**	**TLR9**	-1.3	-1.2	-1.1	1.0	-1.3	-1.1	-1.9	-1.5	-1.4	-3.1	**-3.9**	-2.3	-3.2	-3.0	-1.7

The data represented in Table 3 and Table 2 are from the same experiment, but were separated into 2 different tables for illustration purposes.

(a) normal text = subjectively assigned nominal to low fold change (-3.4 to 3.4).

(b) bold text, negative = subjectively assigned moderate fold suppression (-23.0 to -3.5).

(c) bold text, positive = subjectively assigned moderate to high fold increase (3.5 to 86.0).

(d) text with * = GAPDH, house keeping gene as a reference.

#### Co-stimulatory marker and Fc receptor genes

In addition to cytokine and chemokine gene expression, TLR7, TLR7/8, and TLR9 agonists directly induced the expression of co-stimulatory genes (CD80, CD86, CD40 and CD58) in human B cells (Figure 2 and Table 2). Such markers of activation are considered important for antigen presentation and stimulation of T cells. Additionally, two Fc receptors were modulated by TLR7, TLR7/8, and TLR9 agonists. Specifically, CD23 (FCER2) was up-regulated by these TLR agonists, while CD32 (FCGR2B) was down-regulated. CD23 is an important molecule for B cell activation and growth, as well as being a low-affinity receptor for IgE [25]. CD32 serves as an inhibitory Fc receptor for IgG and appears to co-aggregate with B cell receptor-bound antigen resulting in inhibition of B cell activation [26]. Thus, decreased expression of the CD32 inhibitory gene is associated with heightened antigen presentation, proliferation and antibody production. Interestingly, the degree of modulation for these genes was 2 to 5 times more in CpG2006-treated B cells as compared to B cells treated with 852A, while they were comparable in B cells exposed to 3M-003, which is a more potent activator of TLR7.

#### Proliferation and differentiation

TLR7, TLR7/8, and TLR9 agonists induced mRNA expression of IL21R and myc, and to a lesser extent, fos. Although these genes are associated with differentiation or proliferation, stimulation of B cells with TLR7 or TLR7/8 agonists resulted in minimal proliferation compared to stimulation with CpG2006 (data not shown), which is consistent with a previous report [15]. The anti-apoptotic gene BCL-xL was also upregulated by 852A, 3M-003, or CpG2006, which is consistent with a previous report that the TLR7/8 agonist R-848 enhanced human pDC survival [8]. Induction of anti-apoptotic gene expression in pDC upon stimulation with 852A has also been described [24]. Overall, the data suggest that TLR7- and TLR9-mediated B cell activation engages differentiation, proliferation, and survival pathways within 24 hr after TLR stimulation.

#### Transcription factors

A number of transcription factors and related signaling proteins were also modulated by TLR7- or TLR9-mediated B cell stimulation. NFKB1A, TCFL5, TR3, and DSP2 were up-regulated following TLR7 and TLR9 activation, while GBP2 was down-regulated. Again, the inactive analog 3M-006 and the TLR8 agonist had minimal effects on these transcription factor genes. IKBA (NFKB1A) is one member of the IκB family that functions to inhibit NFκB transcriptional activity (reviewed in [27]). TCFL5 is a basic helix-loop-helix transcription factor whose function is unclear [28]. TR3 is an orphan nuclear receptor that negatively or positively regulates gene expression [29]. DSP2 is member of the dual-specificity-phosphatase family that may negatively regulate STAT3 signaling [30] and may indirectly modulate other transcriptional regulators by inactivating p38 or JNK [31]. In general, TLR7- and TLR9-mediated regulation of the expression of mRNA for various transcription factors and signaling molecules is consistent with the modulation of cytokine, chemokine, proliferation, differentiation, co-stimulatory, Fc receptor and cell survival genes.

### TLR7- and TLR9-mediated cytokine production

**Figure 3:**
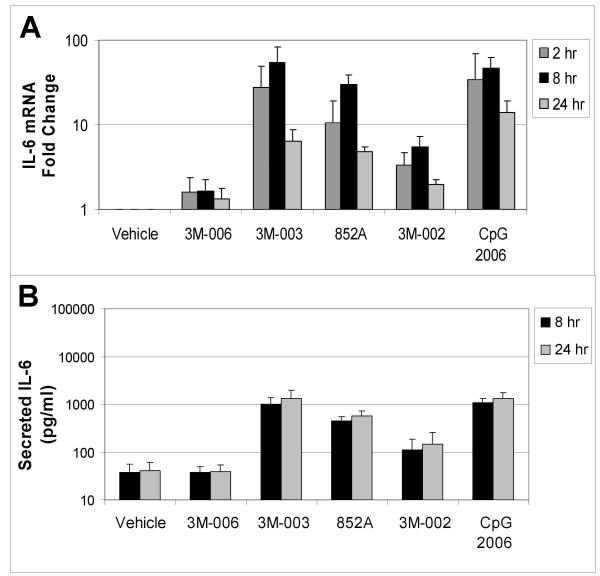
**Changes in mRNA and protein for IL6.** Purified B cells from 3 different donors were stimulated with the indicated IRM or with CpG2006 for 2, 8 or 24 hours, and then were harvested for mRNA analysis. The fold change in gene expression at each time point, normalized to vehicle control, is shown for IL6 (*Panel A*). Conditioned media from the stimulated cells were collected at 8 and 24 hours after stimulation for analysis of protein production. The amount of secreted IL6 (in pg/ml) is shown in *Panel B*. The concentrations of the TLR agonists were: 3M-006, 5 μM; 3M-003, 1 μM; 852A, 3 μM; 3M-002, 5 μM; CpG 2006, 3 μM.

**Figure 4:**
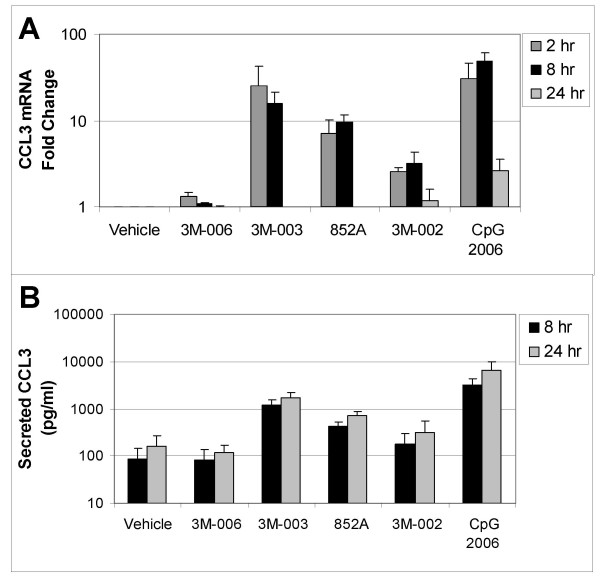
**Changes in mRNA and protein for CCL3 (MIP1α).** Purified B cells from 3 different donors were stimulated with the indicated IRM or with CpG2006 for 2, 8 or 24 hours, and then were harvested for mRNA analysis. The fold change in gene expression at each time point, normalized to vehicle control, is shown for CCL3 (MIP1α), (*Panel A*). Conditioned media from the stimulated cells were collected at 8 and 24 hours after stimulation for analysis of protein production. The amount of secreted CCL3 (MIP1α) (in pg/ml) is shown in *Panel B*. The concentrations of the TLR agonists were: 3M-006, 5 μM; 3M-003, 1 μM; 852A, 3 μM; 3M-002, 5 μM; CpG 2006, 3 μM.

**Figure 5:**
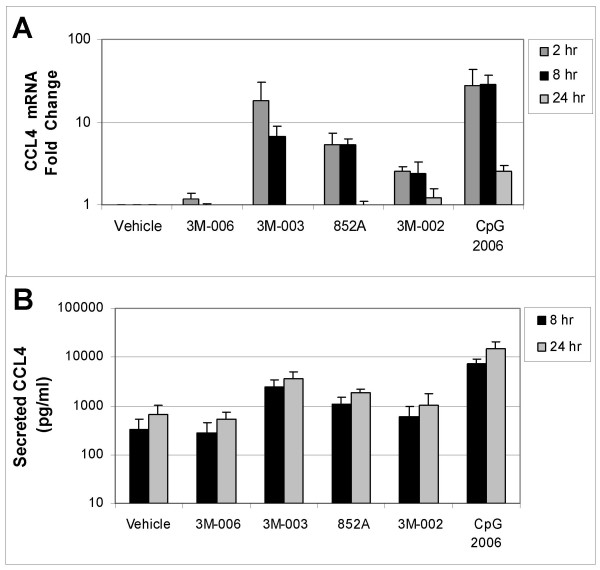
**Changes in mRNA and protein for CCL4 (MIP1β).** Purified B cells from 3 different donors were stimulated with the indicated IRM or with CpG2006 for 2, 8 or 24 hours, and then were harvested for mRNA analysis. The fold change in gene expression at each time point, normalized to vehicle control, is shown for CCL4 (MIP1β) (*Panel A*), Conditioned media from the stimulated cells were collected at 8 and 24 hours after stimulation for analysis of protein production. The amount of secreted CCL4 (MIP1β) (in pg/ml) is shown in *Panel B*. The concentrations of the TLR agonists were: 3M-006, 5 μM; 3M-003, 1 μM; 852A, 3 μM; 3M-002, 5 μM; CpG 2006, 3 μM.

In order to confirm the changes in mRNA expression, induction of secreted protein production from the B cells was measured by multiplex immunoassay. Comparisons were made between different treatments relative to the vehicle control. Figures 3, 4, 5 compare mRNA expression for IL6, CCL3 (MIP1α), and CLL4 (MIP1β) at 2, 8, and 24 hr with protein production at 8 and 24 hr after TLR agonist stimulation of B cells.

TLR7, TLR7/8 and TLR9 agonists induced increases in both gene expression and protein production for IL6, CCL3 and CCL4. Expression of mRNA for all three genes was significantly increased at 2 and 8 hours after stimulation. By 24 hours after stimulation, mRNA for CCL3 and CCL4 had returned to baseline in B cells stimulated with 852A and 3M-003, but was still somewhat elevated in B cells stimulated with the TLR9 agonist. IL-6 mRNA exhibited a different temporal pattern, and was still significantly elevated at 24 hours after stimulation with TLR7, TLR7/8 and TLR9 agonists. Maximal protein production for IL-6, CCL3 and CCL4 was 5 to 50 times greater than the corresponding vehicle control group at 8 and 24 hours after induction by TLR7, TLR7/8 or TLR9 agonists, with 852A exhibiting somewhat less protein production than the other two stimuli. The TLR8 agonist induced a maximum increase of <7-fold in gene expression and < 3 times greater than the corresponding vehicle control in protein production, while 3M-006 induced no significant changes in mRNA or protein for IL-6, CCL3 and CCL4. There was a variable level of secreted IL-8 by treatment with the TLR agonists, ranging from ~20–150 pg/ml. Interestingly, secreted protein levels of IL1β (~50–350 pg/ml), IL2 (~40–60 pg/ml) and IL2R (~20–30 pg/ml) were observed exclusively after treatment with CpG 2006. Other cytokines and chemokines examined (IL1RA, IL4, IL5, IL7, IL10, IL12p40, IL13, IL15, IL17, TNFα, IFNα, IFNγ, GM-CSF, IP10, MIG, Eotaxin, RANTES, and MCP1) were not consistently increased by the TLR agonists (data not shown).

### IRMs and CpG2006 induce IgM and IgG antibody production in human B cells

**Figure 6:**
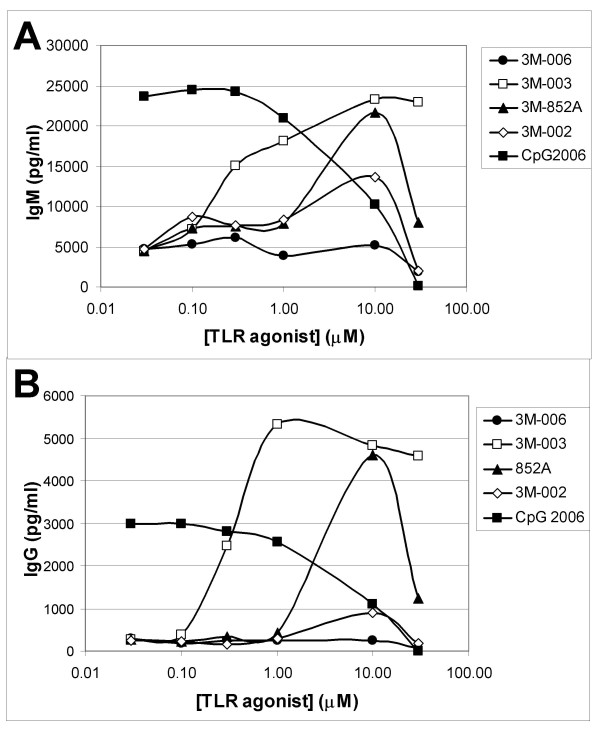
**Antibody production from differentiated B cells.** Purified B cells were cultured for 10 days in the presence of the indicated concentrations of an IRM or CpG2006. Conditioned media from these cultures was analyzed for production of IgM (*Panel A*) or IgG (*Panel B*). Data from a representative donor are shown.

The B cell population in peripheral blood is comprised of naïve, memory and plasma cells. When B cells were cultured for 10 days in the presence of 852A, 3M-003 or CPG 2006, they differentiated, at least in part, into antibody producing cells. Figures 6A &6B illustrate the levels of total IgM and IgG production from B cells treated with various concentrations of one of the IRMs or CPG 2006. B cells cultured with the lowest concentration of CpG tested produced maximal amounts of IgM and IgG. In contrast, higher concentrations of 3M-003 or 852A were required to achieve maximal antibody production. B cells treated with optimal concentrations of the IRMs produced comparable amounts of IgM as those stimulated with the TLR9 agonist. However, they produced significantly more IgG than B cells exposed to CpG2006.

## Discussion

**Figure 7:**
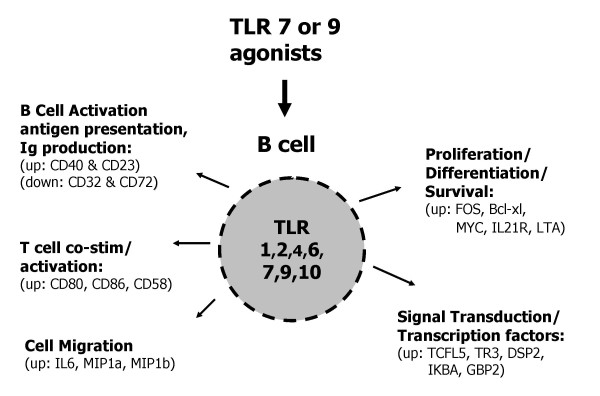
**Functional consequences of TLR7 and TLR9 regulated gene expression in human B cells.** Stimulation of B cells with agonists of TLR7 or TLR9 induces changes in gene expression and protein production that effect B cell function.

In this study, highly purified human B cells (≥99%), containing both naïve and memory populations, were directly activated through TLR7 or TLR9 and changes in gene and protein expression were examined. Overall, there was comparable gene expression and secreted cytokine profile for human B cells treated with the TLR7 agonist 852A (3.0 μM) or the TLR7/8 agonist 3M-003 (1.0 μM) versus the TLR9 agonist CpG 2006 (3.0 μM). TLR7 or TLR9 activation in B cells also induced IgM and IgG antibody secretion in a dose dependent manner. Furthermore, 852A, 3M-003 or CpG 2006 modulated co-stimulatory molecule and Fc receptor expression, thus priming B cells for interactions with other immune cells (e.g. dendritic cells, T cells, etc.). The similarity of gene expression changes induced by TLR7 and TLR9 agonists is not surprising, considering that both receptors use a common signaling pathway mediated by MyD88. A summary of the effects of TLR7 or TLR9 stimulation on human B cell gene expression and function is shown in Figure 7.

The biggest difference between B cell responses induced by TLR7 and TLR9 agonists was observed in immunoglobulin production. Much lower concentrations of TLR9 agonists were able to induce production of IgM and IgG, while peak immunoglobulin production was observed at higher concentrations of TLR7 agonists. The maximal amount of IgM produced by TLR7 or TLR9 agonists was comparable, despite the difference in concentrations needed to induce IgM production. However, TLR7 agonists induced significantly more IgG than TLR9 agonists. The mechanism(s) behind these differences has(have) not yet been elucidated. However, the differential antibody repertoire induced by TLR7 and TLR9 agonists may have a profound effect on an individual's response to infection and long-term immunity.

Despite overall similarity, the induction of expression of mRNA for some genes (e.g. IL6, CCL3 (MIP1α), CCL4 (MIP1β)) was more robust in response to stimulation with CpG 2006 at the dose tested, than with the IRMs that activated TLR7. An additional difference between B cells stimulated with TLR9 or TLR7 agonists was the observation that CpG 2006-treated B cells secreted detectable levels of IL1β, IL2 and IL2R protein, whereas 852A- or 3M-003-treated B cells did not. This could be due to the fact that our pure B cell population contained more naïve cells which respond to CpG and not to TLR7 agonists.

It was reported that naïve B cells express TLR9 and are responsive to CpG 2006, yet do not express TLR7 and are unresponsive to IRMs [15]. That study also showed that type 1 interferons from plasmacytoid dendritic cells (PDCs) are responsible for the up-regulation of TLR7 on naïve B cells, thus enabling their activation by TLR7 ligands such as IRMs [15]. In contrast with naïve B cells, memory B cells exhibited some responsiveness to TLR7 stimulation in the absence of type I IFN, but stimulation of their proliferation was amplified by addition of IFN-α [15]. Although the B cells used in our study were highly purified, no attempt was made to separate the naïve B cells from the memory B cells, and the population used in these studies was not characterized for the relative abundance of these two phenotypes. However, based on published data on young and elderly individuals, the proportion of memory B cells was probably 15–30% [32]. If we assume that the prior report on lack of TLR7 expression by naïve B cells [15] is correct, then the observation that changes in B cell gene expression was directly activated by 852A or 3M-003 in the absence of pDC or type I IFN suggests that the responses monitored in this report were from the memory B cell population. The limited induction of B cell proliferation by the TLR7 agonists is consistent with the previously reported modest stimulation of memory B cells in the absence of IFN-α [15].

The data indicate that CD23 was up-regulated by the TLR agonists, while CD32 was down-regulated, and the degree of modulation for these genes was 2 to 5 times more in CpG2006-treated B cells as compared to B cells treated with 852A, but not 3M-003. This difference could result from the observation that 3M-003 is a more potent activator of TLR7. Changes in these genes may have a significant effect on B cell responses, since CD23 and CD32 are important molecules for B cell activation, antigen presentation, proliferation, and antibody production [25,26]. Thus, it is expected that decreased expression by 852A, 3M-003 or CpG 2006 of the CD32 inhibitory molecule may cause heightened antigen presentation and antibody production.

It has been reported that TLR7-deficient, lupus-prone mice failed to generate antibodies to RNA-containing antigens. TLR9 and TLR7 also had modulatory effects on clinical disease in lupus-prone mice. In the absence of TLR9, autoimmune disease was exacerbated. In contrast, TLR7-deficient mice had ameliorated disease. These findings reveal opposing inflammatory and regulatory roles for TLR7 and TLR9, despite similar tissue expression and signaling pathways. These results have important implications for TLR-directed therapy of autoimmune disease [33,34]. Another study showed that a dual inhibitor of TLR7 and 9, immunoregulatory sequence (IRS) 954, can prevent progression of disease when injected in the lupus prone (NZB×NZW)F1 mice [35]. While it is difficult to reconcile all these findings, it is tempting to speculate that differential expression of TLR7 and TLR9 in naïve vs. memory cells may have an influence on the immune response to self antigens such as RNA.

CpGs were reported to upregulate TRAIL on B cells in PBMC and thereby enhance their ability to kill tumor cells [36]. Evidence also suggests that combinatorial expression of certain cytotoxic TNF family ligands (TRAIL, TNF alpha, Lymphotoxin (LT-α1β2), Fas ligand) on dendritic cells elicits tumoricidal activity [37]. 852A, 3M-003 and CpG 2006 all induced TNF and LTA expression in B cells, which raises the possibly of inducing anti-tumor activity. In addition to its apoptotic effects on certain tumor cells, the secreted form of LTA is believed to play an important role in lymphoctye homing and the development of lymph nodes and spleen [38,39]. Thus, TLR7 or TLR9 agonists may promote anti-tumor activity directly or via other immune mechanisms.

## Conclusion

Our studies demonstrate that human B cells are directly activated by the TLR7 agonist 852A and the TLR7/8 agonist 3M-003 in a similar fashion to the TLR9 agonist CpG 2006. The findings in this report support the utility of 852A or a TLR7/8 agonist like 3M-003 in the treatment of cancer or other conditions in which the activation of B cells may be desirable.

## Methods

### TLR agonists

Small molecule imidazoquinoline TLR7, TLR8, and TLR7/8 agonists: 852A, N-[4-(4-amino-2-ethyl-1H-imidazo[4,5-c]quinolin-1-yl)butyl]methanesulfonamide; formula, C17H23N5O2S; m.w., 361; 3M-002, 2-propylthiazolo [4,5-c]quinolin-4-amine; formula, C13H13N3S; m.w., 243; 3M-003, 4-amino-2-(ethoxymethyl)-,-dimethyl-6,7,8,9-tetrahydro-1H-imidazo [4,5-c]quinoline-1-ethanol hydrate (formula, C17H26N4O2; m.w., 318) and an inactive small molecule TLR7/8 analog, 3M-006, were prepared by 3M Pharmaceuticals. All imidazoquinolines were prepared in DMSO (sterile cell culture grade; Sigma-Aldrich) at a concentration of 10 mM and stored in aliquots at 4°C. Phosporothioate-protected CpG oligonucleotide 2006 (CpG2006, 5'-TCGTCGTTTTGTCGTTTTGTCGTT-3') was obtained from (Genosys, Woodlands, TX).

### Isolation and treatment of human peripheral blood B cells

Human peripheral blood mononuclear cells (PBMCs) were obtained from healthy volunteers from AllCells, LLC. (Berkeley, CA) and Memorial Blood Centers (Minneapolis, MN). PBMCs were isolated using a Ficoll-paque PLUS^® ^(Amersham Biosciences, Piscataway, NJ) density gradient as recommended by the manufacturer. The isolated PBMC were washed once with Dulbecco's Phosphate Buffered Saline without Ca^2+ ^or Mg^2+ ^(Biosource) and resuspended in MACS Running Buffer (pH 7.2 Phosphate Buffered Saline + BSA + EDTA + 0.09% Azide). B cells were enriched using a B cell isolation kit II (Miltenyi Biotech, Auburn, CA) following the manufacturer's instructions. Granulocytes, platelets, erythroid cells and mononuclear cells were labeled with a cocktail of biotinylated CD2, CD14, CD16, CD36, CD43 and CD235a (Glycophorin A) antibodies and subsequently labeled with Anti-Biotin MicroBeads. The positively selected cells were removed using AutoMACS (depletion program performed in triplicate). The enriched, untouched CD19^+ ^population was stained with a cocktail of fluorescently-labeled antibodies (CD19-PE, BD Biosciences, BDCA-4-APC, Miltenyi Biotech) and DAPI (Molecular Probes, Invitrogen Corp, Carlsbad, CA) for dead-cell discrimination and sorted to ≥99% purity on a BD FACSAria by gating on the CD19^+ ^BDCA-4^- ^DAPI^- ^cells (cell purity histogram of 1 representative donor, (see Additional file 1). Note that doublets were removed by first gating on lymphocytes (FSC vs SSC) and then discriminating against doublets on the basis of FSC-A vs SSC-A and then onto SSC-H vs SSC-W. The purified B cells (a mixture of naïve and memory cells) generated by the combination of immunomagnetic bead depletion followed by flow sorting were free of contaminating pDC.

The purified B cells were resuspended in X-vivo 20 medium (BioWhitaker serum free medium, Cambrex Bio Science Walkersville Inc., Walkersville, MD) at 2–3 × 10^6 ^cells/mL and rested 1 hr at 37°C prior to stimulation. For gene expression studies and cytokine and chemokine determination, B-cells were stimulated with 3.0 μM 852A (TLR7 selective), 5.0 μM 3M-002 (TLR8 selective), 1.0 μM 3M-003 (TLR7/8 agonist) or 5.0 μM 3M-006 (inactive analog) dissolved in dimethyl sulfoxide (DMSO, Sigma Chemical) or the same volume of DMSO as the vehicle control, or 3.0 μM CpG 2006 (TLR9 selective) dissolved in tissue-culture grade, endotoxin-free distilled H_2_O. Tissue culture supernatants and cellular mRNA were collected 2, 8, and 24 hour following TLR stimulation. For immunoglobulin determination, purified B cells were cultured in RPMI 1640 with 10% heat-inactivated FBS, 1% penicillin/streptomycin and incubated with 852A, 3M-002, 3M-003, 3M-006 or CpG2006 (0.03, 0.1, 0.3, 1.0, 10.0, 30.0 μM), in 5% CO_2 _for 10 days, at which time the supernatant levels of IgG and IgM were determined by ELISA. Note that TLR selectivity was defined as previous described in genetic reconstitution studies using TLR-transfected HEK293 cells (6). In brief, HEK cells were co-transfected with individual human TLRs and a NFκB-luciferase reporter construct. A TLR agonist was defined as a molecule that could activate NFκB in HEK293 cells that expressed a TLR. Selectivity was defined as the concentration of a given agonist that induced NFκB activation in HEK cells transfected with only one of the TLRs (TLR7, TLR8, or TLR9). As an example, a TLR7-selective agonist activated NFκB at the tested concentration in TLR7 transfected cells, but not in TLR8 or TLR9 transfected cells.

### Cytokine analysis

Tissue culture supernatants were frozen at -20°C and later assayed for cytokines and chemokines using a Human Cytokine Twenty-Five-Plex Antibody Bead Kit for Luminex xMAP™, and Luminex™ 100 System (Biosource, Camarillo, CA and Luminex Corporation, Austin, TX). IgG and IgM were measured by ELISA (Zeptometrics Buffalo, NY).

### RNA stabilization and reverse transcription

RNA was preserved by the direct addition of 400 μl of RLT buffer from the Qiagen RNAeasy kit (Qiagen Inc., Valencia CA) to the cell pellet. Stabilized RNAs were frozen at -20°C until they could be purified using the RNAeasy kits (Qiagen). Purified RNA was then reverse transcribed using SuperScript Double Stranded cDNA Synthesis kit (Invitrogen Corp, Gaithersburg MA), using random hexamer primers.

### Quantitative polymerase chain reaction

Quantitative real-time Polymerase Chain Reaction, RT-PCR was conducted using an open 96 well plate format with ABI validated Taqman^® ^Gene Expression Assays and custom Taqman^® ^Low Density Arrays (Applied Biosystems, Foster City CA). Samples were run in duplicate for the 96 well plate format. PCR cycling conditions were 50°C for 2 minutes, 95°C for 10 minutes, then 35 cycles of 95°C for 15 seconds, and 60°C for 1 minute using an ABI PRIZM 7900HT Sequence Detection System (Applied Biosystems, Foster City CA). Low Density Arrays contained Taqman^® ^reagents for 23 different genes and GAPDH as a reference. Each reagent was run in duplicate for each sample. PCR cycling conditions were 50°C for 2 minutes, 95°C for 10 minutes, then 35 cycles of 95°C for 30 seconds, and 60°C for 1 minute using an ABI PRIZM 7900HT Sequence Detection System (Applied Biosystems, Foster City CA). The instrument software calculated the number of cycles (Ct) required for the accumulated signal to reach a designated threshold value at least 10 standard deviations greater than the baseline. The Ct value is then proportional to the number of starting copies of the target sequence. Relative quantitation of gene expression was performed using the ΔΔCt method as described in User Bulletin #2, PE Applied Systems [16]. The fold modulation was calculated relative to the vehicle control through the same time course. The TLR basal level of gene expression was determined by converting Ct values into a relative copy number using the following assumptions; a copy number of zero is set at 35 cycles, and GAPDH expression as a percentage of total transcripts is consistent from donor-to-donor. The relative copy number was standardized using 2 ng per reaction.

### Gene cluster analysis

Hierarchical cluster analysis was performed with Spotfire DecisionSite-8.1 for Functional Genomics (Spotfire Inc, Somerville, Massachusetts) using the Unweighted Pair-Group Method with Arithmetic mean (UPGMA) and the Euclidean similarity measure.

## Abbreviations

IRM: immune response modifier; ODN: CpG-containing oligodeoxynucleotide; 852A: TLR7 selective agonist; 3M-002: TLR8-selective agonist; 3M-003: TLR7/8 agonist; 3M-006: inactive imidazoquinoline analog.

## Authors' contributions

JAH conceived the study, carried out all of the gene expression experiments and drafted parts of the manuscript. WB, JPV and SSA guided parts of the research and drafted parts of the manuscript. CLR purified B cells to high purity and participated in the cytokine and antibody quantitation assays and data analysis. LN performed the cytokine protein quantitation assays and data analysis. KEL drafted parts of the manuscript and was responsible for the final editing. All authors read and approved the final manuscript.

## Supplementary Material

Additional file 1 Flow cytometric analysis of human B cells. Flow cytometric analysis of human B cells. *Panel A: *B cells were enriched from human PBMC using immunomagnetic beads, and analyzed for purity by flow cytometry using CD-19 and BDCA-4 as markers (pre-sort purity was 80.3%, one representative donor). *Panel B: *The enriched B cell population was then further purified by flow sorting, and analyzed as described above (post-sort purity was 99.9%, one representative donor). Click here for file

Additional file 2 Gene expression profile of human B cells modulated by TLR7, 8, or 9 agonists. Gene expression profile of human B cells from one donor (donor 1) after treatment with TLR7, 8, or 9 agonists. Gene expression was determined at 2, 8 and 24 hours post stimulation. Click here for file

Additional file 3 Interferon and interferon-inducible genes in human B cells treated with TLR7, 8, 9 agonists. Gene expression profile of human B cells from one donor (donor 1) after treatment with TLR7, 8, or 9 agonists. Interferon and interferon inducible genes were minimally and inconsistently modulated by treatment with TLR agonists. Gene expression was determined at 2, 8 and 24 hours post stimulation. Click here for file
