# Supplementary figures and images for: Comparison of human B cell activation by TLR7 and TLR9 agonists
Source: BMC Immunol. 2008 Jul 24;9:39. doi: 10.1186/1471-2172-9-39 (PMC2503978; doi:10.1186/1471-2172-9-39)

## Slide 1
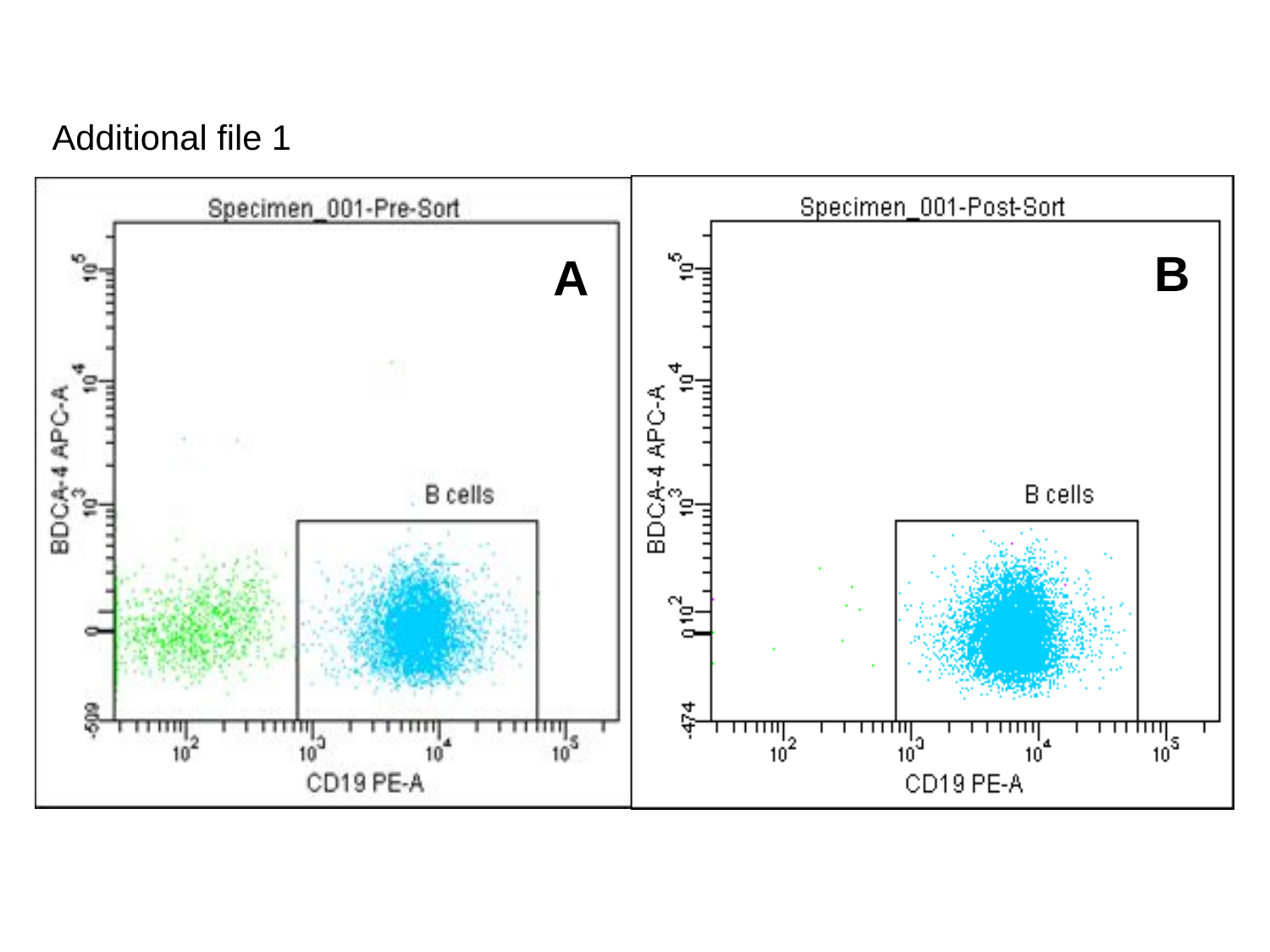

Additional file 1
B
A

Supplement: Additional file 1 — Flow cytometric analysis of human B cells. Flow cytometric analysis of human B cells. Panel A: B cells were enriched from human PBMC using immunomagnetic beads, and analyzed for purity by flow cytometry using CD-19 and BDCA-4 as markers (pre-sort purity was 80.3%, one representative donor). Panel B: The enriched B cell population was then further purified by flow sorting, and analyzed as described above (post-sort purity was 99.9%, one representative donor). [file 1471-2172-9-39-S1.ppt]
