# Supplementary material for: Comparison of human B cell activation by TLR7 and TLR9 agonists
Source: BMC Immunol. 2008 Jul 24;9:39. doi: 10.1186/1471-2172-9-39 (PMC2503978; doi:10.1186/1471-2172-9-39)
Supplement: Additional file 2 — Gene expression profile of human B cells modulated by TLR7, 8, or 9 agonists. Gene expression profile of human B cells from one donor (donor 1) after treatment with TLR7, 8, or 9 agonists. Gene expression was determined at 2, 8 and 24 hours post stimulation. [file 1471-2172-9-39-S2.doc]

**Additional file 2.** Gene expression profile of human B cells modulated by TLR7, 8, or 9 agonists from 1 donor (D1) through a 2, 8, 24 hour time course.

| **Gene** | **Alias** | **3M-006 (D1)** | | | **3M-002 (D1)** | | | **852A (D1)** | | | **3M-003 (D1)** | | | **CpG 2006 (D1)** | | |
| --- | --- | --- | --- | --- | --- | --- | --- | --- | --- | --- | --- | --- | --- | --- | --- | --- |
|  |  | **2h** | **8h** | **24h** | **2hr** | **8h** | **24h** | **2h** | **8h** | **24h** | **2h** | **8h** | **24h** | **2h** | **8h** | **24h** |
| **FCGR2B** | **CD32** | 1.0 | -1.2 | -1.2 | -1.1 | -1.3 | -1.1 | -1.1 | -2.6 | -2.3 | -1.3 | **-7.4** | -2.2 | -1.6 | **-6.7** | **-3.7** |
| **GBP2** | **GBP2** | 1.1 | 1.0 | -1.1 | 1.1 | -1.1 | -1.1 | 1.0 | -2.5 | -2.8 | -1.2 | **-3.5** | -3.1 | 1.1 | -1.2 | -2.6 |
| **CD72** | **Ly-19** | 1.3 | 1.0 | -1.5 | -1.1 | 1.1 | -1.1 | -1.1 | -3.2 | -1.9 | -1.4 | **-5.1** | -1.6 | -1.3 | **-3.5** | -1.4 |
| **GAPDH** | **GAPDH** | 1.0* | 1.0* | 1.0* | 1.0* | 1.0* | 1.0* | 1.0* | 1.0* | 1.0* | 1.0* | 1.0* | 1.0* | 1.0* | 1.0* | 1.0* |
| **IL12B** | **IL12p40** | 1.0 | -1.1 | -1.1 | 1.5 | **8.5** | 1.1 | 1.8 | **9.1** | -2.6 | 3.0 | **16.1** | -2.2 | **5.2** | **11.4** | -1.8 |
| **FCER2** | **CD23** | 1.0 | 1.0 | -1.1 | -1.2 | 1.6 | 1.1 | 1.0 | 3.1 | 1.0 | 1.1 | **7.0** | 2.2 | -1.1 | **3.5** | 1.8 |
| **CCL20** | **MIP3a** | -1.2 | 1.0 | 1.0 | **6.0** | **52.3** | -1.2 | 1.5 | 2.9 | -2.5 | **7.8** | **28.6** | -2.9 | 1.4 | 1.0 | -2.9 |
| **IL-1B** | **IL1F2** | 1.2 | -1.6 | -1.7 | **7.5** | **31.1** | 1.3 | 1.2 | 2.6 | 1.0 | **5.6** | **28.8** | 1.6 | 1.2 | **3.9** | 1.4 |
| **COX-2** | **PTGS2** | 1.1 | -1.2 | -1.1 | **6.7** | **21.7** | -1.3 | 1.4 | 1.0 | -2.7 | **5.2** | **16.8** | -1.8 | 1.3 | **4.9** | **-3.8** |
| **CD86** | **B7-2** | 1.0 | 1.1 | -1.2 | 1.0 | 1.2 | 1.0 | 1.2 | 2.1 | -1.4 | 1.9 | 2.8 | -1.3 | 1.4 | 1.6 | -1.7 |
| **CD40** | **TNFRSF5** | 1.1 | 1.1 | 1.0 | 1.6 | 1.6 | 1.1 | 2.5 | **3.7** | -1.4 | **6.1** | **4.9** | -1.4 | **4.7** | 3.3 | -1.1 |
| **FOS** | **c-fos** | 1.1 | 1.0 | 1.0 | -1.2 | 1.1 | -1.3 | 1.3 | 1.7 | -2.0 | 1.8 | 1.1 | **-3.7** | 1.6 | 1.4 | -2.7 |
| **GOS-2** | **RP1** | -1.2 | -1.4 | 1.2 | 1.6 | 1.6 | 2.3 | -1.1 | 3.1 | 1.0 | 3.2 | **6.3** | 1.8 | 1.9 | **5.3** | 1.5 |
| **NFKB1A** | **IKBA** | 1.0 | 1.1 | 1.1 | 1.2 | 1.9 | 1.1 | 2.0 | **3.8** | -1.3 | **3.7** | **4.8** | -2.0 | 2.8 | **4.0** | 1.1 |
| **TR3** | **TNFRSF25** | 1.1 | 1.3 | 1.1 | 1.0 | 1.2 | 1.1 | 1.5 | 1.0 | -2.0 | 1.5 | -1.8 | -2.7 | 2.0 | -1.5 | 1.0 |
| **CD58** | **LFA3** | 1.1 | 1.1 | 1.0 | 1.3 | 1.2 | 1.0 | 1.7 | 2.0 | -1.3 | 3.3 | 3.1 | -1.2 | 2.4 | 3.2 | 1.0 |
| **IL21r** | **NILR** | 1.2 | 1.3 | 1.0 | 1.2 | 1.7 | 1.1 | 1.7 | **3.5** | 1.1 | **3.6** | **4.9** | 1.3 | 3.1 | **4.0** | 2.3 |
| **MYC** | **c-myc** | 1.1 | 1.1 | -1.2 | 1.2 | 1.6 | 1.1 | 2.1 | 1.6 | -1.3 | **3.5** | 2.0 | -2.8 | 3.4 | **3.6** | 1.6 |
| **CD80** | **B7-1** | 1.2 | 1.2 | 1.1 | 1.0 | 2.0 | 1.3 | 1.4 | **3.9** | 1.3 | 1.5 | **7.3** | 1.4 | 1.5 | **6.1** | 2.0 |
| **TNFa** | **TNFSF2** | -1.2 | -1.2 | 1.1 | 1.7 | 3.1 | 1.3 | **4.0** | **9.5** | 1.1 | **8.2** | 14.9 | 1.0 | **7.5** | **13.8** | **4.7** |
| **IL1a** | **IL1F1** | 0.7 | -1.1 | -1.7 | 2.5 | 2.5 | -1.3 | 2.5 | **4.0** | 1.6 | **4.4** | **5.7** | 1.6 | **3.5** | **4.6** | 1.6 |
| **CCL4** | **MIP1b** | 1.4 | 1.0 | -1.1 | 2.3 | 3.3 | 1.5 | 3.4 | **6.1** | -1.6 | **10.8** | **7.8** | -2.0 | **16.7** | **22.3** | 3.0 |
| **CCL3** | **MIP1a** | 1.2 | 1.1 | 1.0 | 2.2 | **4.5** | 1.5 | **3.5** | **11.4** | -1.3 | **14.4** | **18.9** | -1.4 | **19.0** | **39.3** | **3.7** |
| **BFCL2L1** | **Bcl-xl** | -1.1 | 1.0 | -1.3 | -1.1 | 1.9 | 1.2 | 1.6 | **4.5** | 1.3 | **4.7** | **6.1** | 1.2 | **5.1** | **4.9** | 2.2 |
| **DSP2** | **PAC1** | 1.3 | -1.1 | -1.1 | 1.2 | 1.2 | 1.2 | 2.3 | 2.1 | -1.8 | **4.0** | 2.3 | -2.3 | **3.7** | 1.7 | 2.0 |
| **LTA** | **TNFSF1** | 1.0 | 1.0 | 1.0 | 1.9 | **6.1** | 1.8 | **8.4** | **63.0** | 2.1 | **33.5** | **85.8** | 1.9 | **19.6** | **55.5** | **12.4** |
| **TCFL5** | **E2BP1** | -1.2 | 1.0 | 1.0 | -1.3 | 1.6 | 1.1 | -1.2 | **9.7** | 1.3 | 1.5 | **22.6** | 1.8 | 1.0 | **16.3** | 2.3 |
| **IL-6** | **IFNB2** | 1.4 | 1.9 | 1.3 | 1.9 | **7.0** | 1.9 | **5.2** | **24.9** | **4.6** | **10.6** | **70.8** | **6.9** | **8.3** | **58.9** | **18.4** |

(1) Legend

(a) normal text = subjectively assigned nominal to low fold change (-3.4 to 3.4).

(b) bold text, negative = subjectively assigned moderate fold suppression (-23.0 to -3.5).

(c) bold text, positive = subjectively assigned moderate to high fold increase

(3.5 to 86.0).

(d) text with * = GAPDH, house keeping gene as a reference.
