# Supplementary material for: Comparison of human B cell activation by TLR7 and TLR9 agonists
Source: BMC Immunol. 2008 Jul 24;9:39. doi: 10.1186/1471-2172-9-39 (PMC2503978; doi:10.1186/1471-2172-9-39)
Supplement: Additional file 3 — Interferon and interferon-inducible genes in human B cells treated with TLR7, 8, 9 agonists. Gene expression profile of human B cells from one donor (donor 1) after treatment with TLR7, 8, or 9 agonists. Interferon and interferon inducible genes were minimally and inconsistently modulated by treatment with TLR agonists. Gene expression was determined at 2, 8 and 24 hours post stimulation. [file 1471-2172-9-39-S3.doc]

**Additional file 3.** Interferon and interferon-inducible genes in human B cells were minimally and inconsistently affected by TLR7, 8, or 9 agonists from 1 donor (D1) through a 2, 8, 24 hour time course.

| **Gene** | **Alias** | **3M-006 (D1)** | | | **3M-002 (D1)** | | | **852A (D1)** | | | **3M-003 (D1)** | | | **CpG 2006 (D1)** | | |
| --- | --- | --- | --- | --- | --- | --- | --- | --- | --- | --- | --- | --- | --- | --- | --- | --- |
|  |  | **2h** | **8h** | **24h** | **2hr** | **8h** | **24h** | **2h** | **8h** | **24h** | **2h** | **8h** | **24h** | **2h** | **8h** | **24h** |
| **GAPDH** | **GAPDH** | 1.0* | 1.0* | 1.0* | 1.0* | 1.0* | 1.0* | 1.0* | 1.0* | 1.0* | 1.0* | 1.0* | 1.0* | 1.0* | 1.0* | 1.0* |
| **IFNa-2** | **IFNa-2** | 1.0 | 1.2 | 2.6 | 3.0 | **3.5** | 1.2 | 1.8 | 3.0 | 2.2 | 1.5 | -1.2 | -2.2 | -1.1 | **4.0** | -1.6 |
| **ISG15** | **ISG15** | 1.3 | 1.1 | 1.6 | -1.2 | 1.6 | 1.9 | 1.1 | 1.2 | -1.2 | 1.5 | 3.0 | -1.2 | 1.4 | -1.2 | -1.5 |
| **MX1** | **MX1** | 1.3 | 1.0 | -1.3 | 1.2 | 1.7 | 1.7 | 1.2 | 1.5 | -1.6 | 1.3 | **6.8** | 1.1 | 1.0 | -2.0 | **-3.9** |
| **TLR7** | **TLR7** | -1.1 | 1.5 | -1.1 | 1.2 | 1.2 | 1.2 | 1.3 | -1.3 | -2.8 | 2.6 | -1.8 | -2.9 | 1.6 | -1.7 | **-3.5** |
| **TLR9** | **TLR9** | -1.3 | -1.2 | -1.1 | -1.4 | 1.0 | -1.1 | -1.5 | -1.4 | -1.9 | -3.1 | -1.5 | -1.7 | -3.2 | -1.3 | -1.6 |

(1) Legend

The data represented in Table 3 and Table 2 are from the same experiment, but were separated into 2 different tables for illustration purposes.

(a) normal text = subjectively assigned nominal to low fold change (-3.4 to 3.4).

(b) bold text, negative = subjectively assigned moderate fold suppression (-23.0 to -3.5).

(c) bold text, positive = subjectively assigned moderate to high fold increase

(3.5 to 86.0).

(d) text with * = GAPDH, house keeping gene as a reference.
